# What Color Is Your Anger? Assessing Color-Emotion Pairings in English Speakers

**DOI:** 10.3389/fpsyg.2019.00206

**Published:** 2019-02-26

**Authors:** Jennifer Marie Binzak Fugate, Courtny L. Franco

**Affiliations:** Psychology, University of Massachusetts Dartmouth, Dartmouth, MA, United States

**Keywords:** color, emotion, color-emotion pairings, theories of emotion, data resampling

## Abstract

Do English-speakers think about *anger* as “red” and *sadness* as “blue”? Some theories of emotion suggests that color(s)—like other biologically-derived signals- should be reliably paired with an emotion, and that colors should differentiate across emotions. We assessed consistency and specificity for color-emotion pairings among English-speaking adults. In study 1, participants (*n* = 73) completed an online survey in which they could select up to three colors from 23 colored swatches (varying hue, saturation, and light) for each of ten emotion words. In study 2, different participants (*n* = 52) completed a similar online survey except that we added additional emotions and colors (which better sampled color space). Participants in both studies indicated the strength of the relationship between a selected color(s) and the emotion. In study 1, four of the ten emotions showed consistency, and about one-third of the colors showed specificity, yet agreement was low-to-moderate among raters even in these cases. When we resampled our data, however, none of these effects were likely to replicate with statistical confidence. In study 2, only two of 20 emotions showed consistency, and three colors showed specificity. As with the first study, no color-emotion pairings were both specific and consistent. In addition, in study 2, we found that saturation and lightness, and to a lesser extent hue, predicted color-emotion agreement rather than perceived color. The results suggest that previous studies which report emotion-color pairings are likely best thought of experiment-specific. The results are discussed with respect to constructionist theories of emotion.

## Introduction

The 2015 Pixar movie *Inside Out* is about a girl who has five “basic” emotions living in her head. Each one is colored uniquely (e.g., *anger* is “red”, *fear* is “purple”, and *disgust* is “green”). The idea represented in the movie is that color—just like a set of behaviors, facial expressions, and/or vocalizations—distinguishes one emotion from another. The idea that emotions are real entities, fixed in nature, isn't just a Hollywood invention, however. The idea that emotions are fixed entities with biological “blueprints” has prevailed in Psychology for nearly 150 years (see Gendron and Barrett, 2009 for a history of emotion theory). Despite growing evidence that suggests emotions might be better conceptualized as *nominal* rather than *natural kinds* (Barrett, 2006a,b, 2011, 2012, 2017; Barrett et al., 2007), this idea has not faded quietly. In fact, emotions are so engrained in our world and schemas, nearly every language expresses them *as if* they were fixed entities. In addition, people have strong emotional associations with colors, and even young children have strong color preferences and have specific emotional characteristics that they ascribe to colors (Boyatzis and Varghese, 1994; Zentner, 2001). Certainly such color-emotion pairings fit our folk psychology, as is expressed in our linguistic metaphors. In English, we talk about “feeling blue,” being “green with *envy*,” or “turning red in the face.” But do these color-emotion pairings reflect the structure of emotions themselves? Two different emotion theories (reviewed next) make two very different predictions.

This paper seeks to address whether emotions are specifically and consistently identified with a color within English-speaking samples. Specificity refers to the fact that one (or more) colors distinguish an emotion from another (e.g., a color is specific to an emotion). Consistency, on the other hand, refers to the fact that one (or more) color(s) are chosen reliably for an emotion (e.g., people agree with what color(s) an emotion should be).

Previous research on color and emotion linkages tend to come from exploring peoples' color preferences, or peoples' behaviors toward colored stimuli. The majority of empirical research comes from marketing, art and design, and studies of consumer behavior (e.g., Stone and English, 1998; Singh, 2006; Gilbert et al., 2016). Even those within psychology, however, are not conducted with the structure of emotion as the focus^1^. While this research is undoubtedly valuable (some of which is reviewed below), the current study is the first to use emotion theory to explore whether colors are diagnostic of emotion categories.

According to one widely-held view of emotion, people experience and perceive emotion because people *have* emotions. According to many individual theories within this view, emotions are treated as biological distinct entities, each with a separate internal mechanism that gives rise to a suite of coordinated and prescriptive reactions that define the emotion and make it different from another emotion (e.g., behavior, facial expressions, tone of voice, bodily changes, brain changes) (Tomkins, 1962, 1963; Izard, 1971, 1994; Ekman, 1972, 1992, 1993; Scherer, 2000; Matsumoto et al., 2008; Brosch et al., 2010; Ekman and Cordaro, 2011; Sauter et al., 2011). As a result, a person is able to measure behavior, facial muscle activity, vocal acoustics, autonomic physiology, or brain activation and know what emotion it is that (s)he or another person is experiencing. Among such emotions are those in English we call *anger, fear*, etc. (Ekman, 1992). Because in these theories emotions are thought of as biological distinct entities, their signaling behavior is thought to provide an honest and viable cue of the sender's internal state (which should predict his or her behavior and the like). Accordingly, an emotion would be associated with a particular color because the color is reflected in a biological way. Color vision evolved because it afforded survival since it likely added fitness-relevant behaviors (Hutchings, 1997; Byrne and Hilbert, 2003). For example, the color “red” is directly correlated with the level of testosterone in males across many species (e.g., high-ranking males often show more bright coloration). Accordingly, “red” would likely portray potential threat and increased likelihood of aggression associated with rank. Behavioral research reinforces this notion, including increasing threat perception and dominance (Hill and Barton, 2005; Feltman and Elliot, 2011; Elliot and Maier, 2012; Young et al., 2013). Colors such as olives and browns are most likely to convey rotting food or feces which should be avoided, and should therefore induce a feeling of *disgust*. That is, the specific colors assigned to an emotion should be based on an evolutionary honest signal or should at least promote evolutionary success (Mollon, 1989; Palmer and Schloss, 2010).

Biologically-mediated relationships between wavelength of color and arousal have long been hypothesized, and mainly conform to the Yerkes-Dodson law of arousal-performance (Yerkes and Dodson, 1908). For example, long wavelength colors (e.g., “red,” “orange,” “yellow,” etc.) are more arousing than short wavelength colors (Lewinski, 1938; Goldstein, 1942; Wilson, 1966), and therefore should increase performance. Likewise, pleasure is associated with brighter, more saturated colors, with the relationship tending to be curvilinear (Guilford and Smith, 1959). A few studies provide evidence for specific colors affecting the body's physiological responses to arousal (i.e., skin conductance, heart rate, and respiration) (e.g., Wilson, 1966; Jacobs and Hustmyer Jr, 1974), yet other studies have found reverse effects of color on arousal or no effects at all. Other studies provide evidence that physiological effects of colors are cognitively mediated (e.g., Kaiser, 1984; Detenber et al., 2000).

It is also possible that biologically-derived color-emotion associations might be modified, reinforced or conventionalized as a result of frequently-paired associations. That is, initial tendencies can be exaggerated by the constant associations of color-emotion within a culture. From a very young age, people are constantly exposed to explicit and implicit pairings, and once these pairings are learned they can be associated with knowledge and behaviors outside the realm of consciousness (Bargh, 1990; Elliot et al., 2007). They can also serve as unconscious primes to automatically influence cognitive processing. Put into practice, “red” and its association with danger, is reinforced in our culture by the “red” of stoplights and stop signs, the “red” of fire alarms, the “red” of pens used to correct papers, etc. (Elliot et al., 2007).

Other theories of emotion, however, do not treat emotions as biological entities, but rather explanatory constructs that a person evokes to explain the primitive changes in his or her own internal sensations or such similar changes within another person (Russell, 1980; Barrett, 2006a,b). In doing so, the individual arrives at a diagnostic category in his or her body (or in another's body) using situational cues and his or her conceptual knowledge. According to one prominent theory (Barrett, 2006a,b, 2011, 2012, 2017), an emotion is *created* when a person uses his or her conceptual knowledge to label (with an emotion word) these internal sensations to arrive at the constructed classification of a particular emotion^2^. Consequently, specific color-emotion associations should be based on a person's beliefs as experienced in their culture and through their language, rather than on evolutionary honest signals.

### Survey Evidence for Color-Emotion Associations

Despite the plethora of information available on the internet, there is little empirical research for consistency and specificity of color-emotion pairings. Those studies which have explored such relationships tend to suffer from several methodological restrictions, and/or do not look at emotions as the source of investigation. Other studies ask participants to rate colors on a list of emotional adjectives, which only loosely map onto some aspects of emotion while blurring other defining features.

In the first empirical study of color and emotion, English-speaking participants selected one of eight colored pieces of paper for each of several emotion adjectives. Participants chose “black” and “brown” for adjectives related to *sadness*, and “yellow” for adjectives related to *happiness* (Wexner, 1954). Despite these pairings, none of the colors accounted for more than 50% of the answers, suggesting a high amount of variability among respondents. In another classic study, English-speaking children and college students chose “yellow,” “orange,” “green,” and “blue” crayons to color *happy* images, but “red,” “brown,” and “black” to color *sad* images (Cimbalo et al., 1978).

More recently, English-speaking participants saw 13 colors (including “black”, “white”, and “gray”) and gave one emotional adjective for each color (Kaya and Epps, 2004). The majority of participants produced emotional adjectives related to *anger* when viewing “red,” adjectives related to *calmness* when viewing “blue,” those relating to *fear* when viewing “black,” those relating to *happy* when viewing “yellow,” and adjectives related to *disgust* for the color “green-yellow.” These findings are somewhat, but not fully, consistent with another study of English-speaking participants. Here, participants assigned “red” most often to *anger*, “green” to *jealousy*, and “yellow” to *happy*, and “blue” to *sad* (Sutton and Altarriba, 2016). Yet “red” was also the most frequent color listed for *contempt, fear*, and *surprise*; and “green” was also the most frequent color for *disgust*; “yellow” also for *joy*; and “blue” also for *pride*. Therefore, in both studies there were no consistent and specific pairings.

Despite the generalized lack of inferred consistency and specificity, there are several other major problems with all of these studies. First, some use color words rather than color squares (actual colors). Obviously, what “red” a person thinks of when asked to associate it with anything (emotion or otherwise) is going to vary widely. Second, even the studies which do use color squares fail to control or recognize that perceived color is determined by hue, saturation (chroma), and brightness/lightness (HSLs) (Centore, 2012). Therefore, one hue may be seen as several different “colors,” depending on the saturation and brightness. In addition, the space of color is not spherical, but rather lopsided and a partially flattened globe. The result is that the maximum saturation for a hue near “bluish-green” is not nearly as extreme as the maximum saturation for hues near “red” (see D'Andrade and Egan, 1974). Many of the color-emotion findings might therefore be due less to hue, but rather to the degree of saturation and brightness (Whitfield and Wiltshire, 1990). For example, “yellow” might be rated as *happy* because it is often imagined (or depicted) at full saturation and brightness (e.g., a sunny “yellow”). A mustard “yellow” at the same hue—but one that is low in the other two properties—is unlikely to be very *happy*.

Only one study has tried to explain how hue, saturation, and brightness contribute directly to the *emotional* dimensions of pleasure, arousal, and dominance (Valdez and Mehrabian, 1994). In that study, brightness (69%) and saturation (22%) predicted pleasure, whereas saturation (60%) and darkness (31%) mainly predicted arousal. Dominance was mainly predicted by brightness (and to a lesser extent saturation).

### Summary of Previous Studies

Although several studies associate colors with emotions (see Table 1), the data are far from clear. Undoubtedly, the strongest link between an individual emotion and color is “red” and *anger*, which has been noted across studies and formats (e.g., Kaya and Epps, 2004; Sutton and Altarriba, 2016). Yet, none of these studies show that “red” is specific to *anger*, as it has also been associated with *love* and *embarrassment*, especially when the range of emotions is restricted. There is less evidence (although some studies report a link) for “yellow” and *happiness*. Yet, “yellow” has also been associated with other emotions, including *envy* in some cultures. Furthermore, a handful of studies report a link between “blue” and *sadness*, but other studies show that “blue” is associated with *calmness*. In addition, behavioral work shows that some color-emotion pairings might be unconscious and can be modified by exposure to learned behaviors (e.g., Elliot et al., 2007). Finally, the brightness of a color seems to reflect a dimension of “activity” or “pleasure,” whereas the saturation of a color reflects a dimension of “potency” or “arousal.”

**Table 1:** Summary of previous color-emotion studies by date.

**Author(s)**	**Participants**	**No. of Colors/Emotions**	**General results**	**Consistency/Specificity?**
Wexner, 1954	English-speaking adults	Eight colored pieces of paper; matched to 11 emotional adjectives	“red” = related to *exciting* and *protective;* “black” and “brown” *=* related to *sadness*; “black” = related to *powerful* “yellow” = related to *cheerful;* “orange” = related to *distressed;* “blue” = related to *tender* and *secure* and *calm;* “purple” = related to *dignified*	Not tested
Adams and Osgood, 1973	Male high school students in 23 countries	Eight color words rated on 12 opponent word scales	Across countries: “Blue” and “green” *= cool;* “red” and “yellow” = *warm;* Differences across cultures noted but not discussed	Not tested
Cimbalo et al., 1978	English-speaking children	Used seven crayons to color eight pre-determined happy and sad images	“yellow,” “orange,” “green,” and “blue” = *happiness;* “red,” “brown,” and “black” = *sadness*	Not tested
Hupka et al., 1997	Participants in five countries (Germany, Mexico, Poland, Russia, and the US)	Twelve color words associated with four emotions	*Anger* = “red” and “black” (Germans, Mexicans, Russians, US); “red” and “black” and “purple” (Poles); Envy = “black” and “yellow” and “purple” (Russians); “yellow” (Germans); “black” and “purple” (Mexicans); “black” and “red” and “green” (US); “black” and “red” (Poles); Fear = “black” (Germans, Poles, Russians, US); “red” and “black” (Mexicans) Jealousy = “red” and “black” (Russians, Mexicans, US); “red” and “yellow” (Germans), “red” (Poland)	
Madden et al., 2000	Adults in Australia, in Brazil, Canada, Columbia, Hong Kong, People's Republic of China (PRC), Taiwan, and the US	Ten colors on computer screen rated on 20 opponent word scales (only some emotional)	Across countries: “green”/“blue”/“white” *= gentle; calming; peaceful* (some countries also added in *beautiful* and *pleasant)* “black”/”brown” *sadness; stale* (some countries also added *formal* and *masculine)* “red” = *active; hot; vibrant* (some countries also added *pleasant)*	Not tested
Kaya and Epps, 2004	English-speaking adults	Thirteen colors on computer screen and stated how each color felt	Only emotions same as ours listed: (results in next column) *Anger Annoyed Bored Calm Disgust Excited Fearful Happy Loved Sad*	Inferred Consistency: Top color % > twice second color: *Anger* = “red”; *Bored =* “gray”; *Calm =* “blue”; *Fear* = “black”; *Happy =* “yellow”; *Disgust* = “green-yellow”; Inferred Specificity from potentials above: Top rated emotion % > twice emotion (or > 50%): “yellow” = *Happy* “blue” *= Calm*
Gao et al., 2007	Adults in Japan, Thailand, Hong Kong, Taiwan, Italy, Sweden, and Spain	Two hundred and fourteen color samples rated on 12 opponent word scales	Across cultures: 2 Major factors (82% overall variance): Factor 1 = chroma (saturation) = activity/excitement Factor 2 = lightness = potency/forcefulness Differences across cultures on emphasis of HSL: chroma more important for Italians and HK; hue for Japanese, Taiwanese, Swedish, and Spanish; chroma and hue equally important for Thai.	Not tested
Sutton and Altarriba, 2016	English-speaking adults	One hundred sixty emotional items and listed a color	Only emotions same as ours listed: (results in next column) *Anger Contempt Disgusted Embarrassed Fear Happy Jealousy Joy Love Sad Proud (Pride) Surprise Shame*	Inferred Consistency: Top color % > x2 second color (or >50%): *Anger =* “red” *Embarrassed =* “red” *Happy =* “yellow” *Jealousy* = “green” *Love =* “red” *Sad* = “blue” Inferred Specificity from potentials above: Top rated emotion % > x2 emotion (or > 50%) “green” = *jealousy* “blue” = *sad* “yellow” = *happiness*
Hanada, 2017	Japanese-speaking adults	Forty colors on computer screen and stated how each color felt	“red” = *Active emotions (e.g., anger, passion, excitement);* “orange” and “yellow” = *Steadier emotions (e.g., pleasantness, happiness);* “dark blue” and “violet” = *Inactive and negative emotions (e.g., disgust, hatred, depression, and fear)*	Not tested

### The Current Studies

The purpose of this research was to test whether English-speaking North Americans show consistency and specificity among color-emotion pairings when a variety of controlled color stimuli (i.e., color swatches) are used. In both studies, participants (*n* = 73 and *n* = 52) completed an online survey in which they could chose up to three colors to associate with a list of emotion words. Study 2 was a replication of study 1, but added in an additional 10 emotions for a total of 20 emotions. In addition, we added or changed 12 colors to better represent color space (hue, saturation, and light) for a total of 28 colors. The rationale for study 2 was 4-fold: to address the criticisms that (1) study 1 was underpowered, (2) that more emotions should be investigated, (3) the choices of color should be better distributed with respect to HSL, and (4) that participants' perception of colors might be different across different devices. To this last criticism, we also conducted an additional control study in which a separate group of participants (*n* = 25) completed the same survey under controlled lighting conditions on one color-calibrated monitor (see Survey Presentation: Additional Laboratory Control).

In both studies, participants could chose up to three colored squares for each emotion and then indicated a numerical value as to the strength of that relationship using a 10 point Likert scale. In study 1, participants were also asked to pick only one, different color for each emotion to assess the differences in a restricted vs. less-restricted setting. Results for this “forced choice” question are discussed in Supplemental Materials.

In agreement with theories of emotion which are constructed, we predicted that there would be few instances in which participants agreed with one another on what color best depicted an emotion (i.e., consistency), and few unique pairings between an individual color(s) and an emotion (i.e., specificity). In addition, we predicted that any positive results would not hold up under statistical re-sampling or with a set of participants with minor changes in the procedure (Hypothesis 1). This would suggest that effects which have been previously reported are experiment-specific and not generalizable across studies when different methodologies or participants are used.

Finally, based on some suggestion from previous literature (Valdez and Mehrabian, 1994), we predicted that saturation and lightness would be better predictors of the colors people associate with an emotion than perceived color (Hypothesis 2).

Our research is novel in that it uses two theoretical tenets inherent to emotion theory, which we precisely operationalize and test statistically. In addition, we present a large list of emotions, present our colors as swatches rather than color words (paying attention to HSL), and allow participants to indicate a strength of agreement for up to three colors for each emotion. We also compare people's agreement using this format with a more traditional format in which participants were asked to choose one color for each emotion. Finally, we bootstrapped our data in study 1 to show that the likelihood of replicating any individual color-emotion pairing was well-below the accepted level of statistical significance. Study 2 confirmed the fact that any consistent and specificity findings which occur are not stable even among English-speakers.

## Methods

### Participants: Study 1 and 2

In study 1, 74 English-speaking adults completed the online color-emotion survey. One participant did not complete the survey and their data were removed. Participants were recruited from the general public via targeted email and then through “snow-ball” sampling to complete the survey online without identifying information. These participants were not compensated. The survey was available on Facebook and the PI's personal webpage for a total of 9 months. All participants identified as native English-speakers with high command of the English language and resided in either America or Canada. Five of the 74 participants had additional fluency in another language. Among the 74 participants, 17 were recruited from the sponsoring University. These participants received one research credit as part of an introductory psychology class.

In study 2, 104 native English-speakers from the United States, Canada, and India completed a similar version of the online survey, through Amazon Mechanical Turk (M-Turk)—a crowdsourcing dissemination apparatus. Participants were required to be “master-Turkers” and completed the survey on an iPhone in which the screen brightness could be controlled (see Survey Procedure). They received $1.00 in their Amazon account once they completed the survey. Forty-eight native English-speakers who completed the survey in English had additional fluency (indicated as above a “5” of 10 on the scale) in another language: Tamil (*n* = 26), Arabic (*n* = 1), Bengali (*n* = 3), Hindi (*n* = 9), Vietnamese (*n* = 1), German (*n* = 1), and French (*n* = 7). In this study, we removed participants with additional high fluency in another language(s). Four participants did not complete the survey and their data were removed. Fifty two participants' data were analyzed. Forty-four percent of the remaining participants were males, and the majority (29%) were between 25 and 34 years old.

In the laboratory control study, an additional 25 participants from the sponsoring University completed the same survey under controlled lighting conditions on one color-calibrated monitor. These participants received one research credit as part of an introductory psychology class. Both studies, as well as the laboratory control study, were carried out in accordance with the recommendations of the University of MA–Dartmouth, IRB # 14.051. The protocol was approved by the A. Karberg, Director of Institutional Compliance. All subjects gave online informed consent in accordance with the Declaration of Helsinki.

### Survey Presentation

The online survey was similar for both studies 1 and 2. The survey in study 1 was programmed in HTML by an outside programmer and housed on the first author's personal, secure internet domain. It was made available by link on the first author's professional website and on Facebook. The survey in study 2 was compiled using Qualtrics because it was easily integrated into M-Turk. The survey was advertised on M-Turk in order to recruit English-speaking participants from a larger demographic in a shorter period of time.

Both surveys began with directions and informed consent (which was obtained by checking a box to continue). In addition, in study 2, participants received a visual diagram of how to adjust the brightness on their screen to 100% and to temporary remove “night mode” viewing from their iPhone. These two small changes helped to control for screen brightness which otherwise might have differed in study 1, and affected how participants perceived each color.

Participants in both studies were then asked the same five demographic questions (age, biological sex, country of origin, fluency in English, and fluency with additional languages). Participants in study 1 advanced the survey page to next see a list of 10 emotion words (presented randomly across participants) with 23 colored squares (programmed in random order, but not randomized across participants) underneath the list of emotion words. The color swatches totaled roughly one-half of the page and were of equal sizes, so that participants (regardless of viewing device) could see the full list of emotion words and colors on one page without scrolling. Participants moved one colored square to the box next to each of the emotion words (for results of this question, see Supplemental Materials). Participants could only use each color once, and therefore not all colors were used.

Next, participants in both studies received the same list of emotions (in random order for both studies) with a matrix of all the colored squares (programmed in the same, but random order) below each emotion word. The color squares comprised a grid in which each color was the same size. Participants could choose (by clicking) on as many as three colors. Once a color was clicked, a Likert scale (1 = a small amount, 10 = a lot) appeared prompting them to indicate the strength of this color-emotion relationship. Participants did not need to select three colors (at minimum they needed to select one color), and could repeat numeric values.

Participants in both studies then completed a color preference checklist and finally labeled the colors of the swatches (at the survey's end). The results of these questions are not reported in this paper. Participants could not go back once a page was completed, and the four basic pages of questions (demographics, multiple choice intensity, preference, and labeling) for both studies came up in the same order for all participants. The average time to complete the survey was 15 min in both studies. All participants were required to respond to every question or the survey would indicate a missing response.

#### Additional Study 2 Control (Laboratory Study)

All participants completed the survey in the first author's laboratory, in which the ambient lighting was controlled and the computer monitor was calibrated for each participant^3^.

### Selection of Colors: Study 1

We considered systematic variations in color, as well as color terms in English, and past research. We used 10 Munsell hues (R, YR, Y, GY, G, BG, B, PB, P, RP). We chose roughly two different lightness values and saturations at each hue. We also included three achromatic colors: white, black and gray. Finally, we chose the Hex Color closest to the Munsell color that was considered “web safe,” meaning that across participants' devices, colors would appear the same. We use the common name for the colors in this paper, but provide HSL, RGB, CIE (Commission Internationale de l'Éclairage) (CIE, 1932), and HEX equivalents (see Appendix A).

### Selection of Colors: Study 2

We kept 16 colors from the original 23 colors from study 1. We removed seven colors (chocolate, jade, sky blue, indigo, aqua, turquoise, and violet) since they were rarely selected in study 1 or were slightly different from the colors reported in Hanada (2017), which was published between the time of study 1 and study 2. We added 12 colors from previous studies: four colors from Kaya and Epps (2004)—teal, purple, bright blue, periwinkle—and six colors from Hanada (2017)—brown, light yellow, dark green, light blue, dark blue, and dark purple. Finally, we added two additional colors never used in research (dark red and light orange) to compile an exhaustive list of all colors across the spectrum. Overall, the 28 colors comprised ten saturations (chroma), 20 lightnesses (bright/dullness), and 23 hues.

### Selection of Emotion: Study 1

We chose ten different “basic” emotions to list as words. Although some researchers identify six basic emotions, others recognize more (e.g., 10 or 12, Plutchik, 1980). The final emotions were: *anger, calmness, contempt, disgust, fear, envy, happiness, jealousy, sadness*, and *surprise*.

### Selection of Emotion: Study 2

For study 2, we kept all original emotion words, but included an additional ten emotion terms. These terms were recently considered as “basic” by Cowen and Keltner (2017). The added emotions were*: alert, awe, boredom, disappointed, empathy, guilt, joy, love, pride*, and *shame*.

## Results

### Consistency: Study 1

We identified the top-three indicated colors (for each emotion) by frequency. To test for consistency of a color with an emotion, we asked whether the intensities of these top-indicated colors differed for an emotion using non-parametric Freidman's tests. We also reported Kendall's *W*, which indicates agreement of raters. Kendall's *W* is linearly related to the mean value of Spearman's rank correlation coefficient, but makes no assumptions regarding the nature of the probability distribution and can handle any number of distinct outcomes (Kendall and Babington Smith, 1939). We report analyses for consistency which did not reach statistical significance in Supplemental Materials. Alpha for all analyses was set to 0.05.

Finally, we asked whether the intensities of top colors would differ from those given to other emotions if we were to resample our data. This is especially important, as null hypothesis testing based on single distributions has many pitfalls and is slowly being moved away from in social sciences (see Cumming, 2014 for a review; see also Kline, 2013). In fact, APA now recognizes re-sampling (in which bootstrapping is one technique, e.g., Efron and Tibshirani, 2001) as a preferred method in research and should be used when possible. To do this, for each emotion we created a three-color matrix by intensity and then performed a pairwise non-parametric bootstrap for the probability that the intensity of the color on the row was greater than the color on the column of the matrix. The number of samples which went into the bootstrapping process was 10,000^4^.

#### Anger

**Figure 1:**
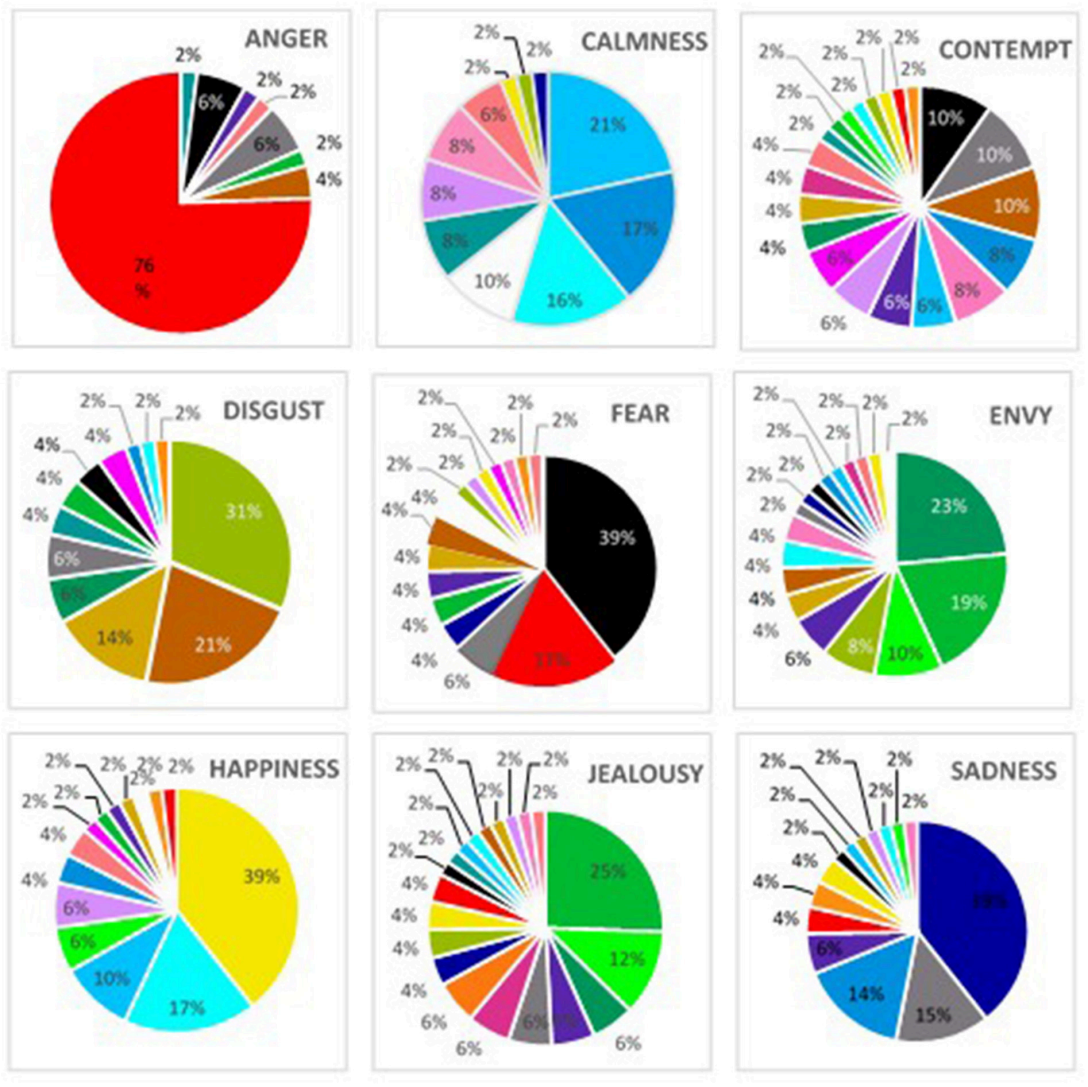
Frequency of selection of colors for each emotion: study 1. Colors represent the actual color swatches. Labels for these colors are used in the next table. See Appendix A to associate the color name with each color.

“Red” was the most frequently chosen color, followed by “black” and then “gray” (Figure 1). The intensity for the color “red” was high, whereas the intensities for “black” and “gray” were moderate and low, respectively (Table 2). The intensities among the three colors differed, χ^2^_(2, N = 73)_ = 97.87, *p* < 0.001, as did the intensities among all three colors at follow-up, *p* < 0.001 level. There was high agreement among raters, Kendall's *W* = 0.670. While these analyses suggest that “red” *might be* specific to *anger*, resampling the data showed that the probability of replicating this finding would be < 95%: We found that the probability of selecting a sample in which the intensity of “red” exceeded that of “black” was around 60% (CI = −4:7) ^5^, whereas the probability of selecting a sample in which the intensity of “red” exceeded that of “gray” was about 78% (CI = −3:6). The probability of selecting a sample in which the intensity of “black” exceeded “gray” was about 57% (CI = −5:6). Therefore, on average, we would expect to find the intensity of “red” to exceed that of “black” 60% of the time. Using an alpha of 0.05 as a level of statistical significance would require finding the effect 95% of the time. Clearly, this falls short.

**Table 2:** Raw mean intensity (followed by SD) of the top colors selected for study 1.

**Coloremotion**	**“Black”**	**“Red”**	**“Gray”**	**“Yellow”**	**“Light Purple”**	**“Sky Blue”**	**“Jade”**	**“Green”**	**“Aqua”**	**“Indigo”**	**“Blue”**	**“Bright Pink”**	**“Chocolate”**	**“Dark Yellow”**	**“Light Green”**
Anger	4.5 4.2	8.6 2.7	1.0 2.6	X	X	X	X	X	X	0.5 1.9	– –	X	X	X	X
Calmness	X	X	X	X	2.3 3.7	3.1 4.4	X	X	1.6 3.4	X	2.4 3.7	X	X	X	X
Contempt	1.7 3.4	X	1.0 2.5	X	1.2 2.9	X	X	0.5 1.9	X	X	0.5 2.0	0.6 2.0	0.7 2.1	0.8 2.4	X
Disgust	X	X	X	X	X	X	0.5 1.9	0.5 1.7	X	X	– –	– –	3.6 4.1	3.2 3.7	3.1 3.9
Envy	X	1.7 3.3	X	X	X	X	2.7 4.1	3.0 4.2	X	X	– –	X	X	X	1.4 3.1
Fear	5.7 4.3	2.5 3.7	1.6 3.2	1.0 2.7	X	X	X	X	X	0.9 2.5	X	X	X	X	X
Happiness	X	X	X	5.3 4.6	X	2.6 4.0	X	X	2.3 4.0	X	0.6 2.2	1.4 3.0	– –	X	X
Jealousy	X	2.6 3.7	X	X	X	X	2.4 4.0	2.3 4.0	X	X	–	X	X	X	1.4 3.2
Sadness	2.4 4.0	X	4.2 4.2	X	X	X	X	X	X	3.4 4.0	X	X	0.8 2.3	X	X
Surprise	X	X	X	2.6 3.9	0.9 2.3	0.6 2.1	X	X	2.1 3.4	X	X	2.6 3.9	– –	X	– –

*Only colors listed which were among the top three for any emotion are listed. X indicates not compared because color was not among the top three by frequency for that emotion*.

#### Fear

“Black” was the most frequently picked color, followed by “red” and then “gray” (Figure 1). “Black” had a high intensity, whereas “red” and “gray” had low intensities (Table 2). The intensities among the three colors were significant, χ${}_{\left(2,\text{N}=73\right)}^{2}$ = 38.82, *p* < 0.001, and the intensities between “black” and “red” were also significant, *p* < 0.001 level. There was only low-moderate agreement among raters, however, Kendall's *W* = 0.266. Again, resampling our data showed that this was an unlikely result. The probability of selecting a sample in which the intensity of “black” exceeded that of “red” was about 54% (CI = −5:8), whereas obtaining a sample in which the intensity of “black” exceeded that of “gray” was only slightly higher, 62% (CI = −5:8). Therefore, despite the fact that the intensity of “black” was high and differed from that of “red,” resampling the same data suggested that we would find black to be consistently paired with fear about half the time.

#### Happiness

“Yellow” was the most frequently picked color, followed by “sky blue” and then “aqua” (Figure 1). “Yellow” had a high intensity, whereas “sky blue” and “aqua” had more moderate intensities (Table 2). The intensities among the three colors were significant χ^2^_(2, N = 73)_ = 18.78, *p* < 0.001, as was the difference between “yellow” and “sky blue,” *p* < 0.001. There was low-moderate agreement among raters, however, Kendall's *W* = 0.129. Again, bootstrapping showed that this effect was unlikely: The likelihood that we would obtain a sample in which the intensity of “yellow” exceeded that of “sky blue” and “aqua” was about 51% (CI = −3:6) and 44% (CI = −3:8), respectively. All other probabilities from resampling the intensities of the top three colors were < 50%. Therefore, despite intensity analyses which showed that the intensity of “yellow” differed from that given to the other colors, re-sampling our data suggested that this would be likely about 50% of the time.

#### Sadness

“Gray” was the most frequent color indicated for *sadness*, followed by “indigo” and then “black” (Figure 1). The intensities for all three colors were moderate (Table 2). The intensities among the three colors differed only marginally, χ^2^_(2, N = 73)_ = 5.82, *p* = 0.05. Despite this, the agreement among participants was extremely low, Kendall's *W* = 0.040. This was confirmed by the low probabilities of replicating this result when resampled our data: All resampling probabilities were < 46%. Therefore, we conclude that “gray” is not likely to be consistently paired with *sadness*.

#### Summary Consistency

Four of the 10 emotions showed statistical evidence for consistency, yet agreement among raters in all conditions was low to moderate. The exception was “red” with *anger*, in which the agreement was relatively high. It is also worth noting that when we analyzed the frequencies from the “forced choice” question (see Supplemental Materials), we found statistical evidence for three of the four emotions listed above. The only pairing which did not replicate was *sadness*.

Yet, when we resampled our data, even “red” being selected as the most intense color for *anger* was not likely to replicate: Rather we would expect this effect to occur about 60% of the time, well below the 95% used in statistical significance testing. These numbers are reflected in the discrepancies among the color-emotion literature. Individual studies that do suggest consistent pairings typically restrain participants' choices and/or use categorical assignments (e.g., yes, no). Study 2 shows that this is the case, as when the context of the study changes (by adding more emotions and color), these consistency pairings do not replicate.

### Specificity: Study 1

We followed similar logic as above, except that we performed the analyses by color (rather than by emotion). Four of the 23 colors were shared among the top three choices for at least two emotions. An additional five colors were only selected among the top three for one emotion, but we tested them against the second and third most frequently chosen emotions (e.g., “indigo,” “bright pink,” “chocolate,” “dark yellow,” and “light green”). We did not analyze the remaining colors because they were never in the top three for any emotion, and participants rarely chose them. We analyzed each color separately. We report colors that did not meet statistical significance in Supplemental Materials.

#### “Red”

**Figure 2:**
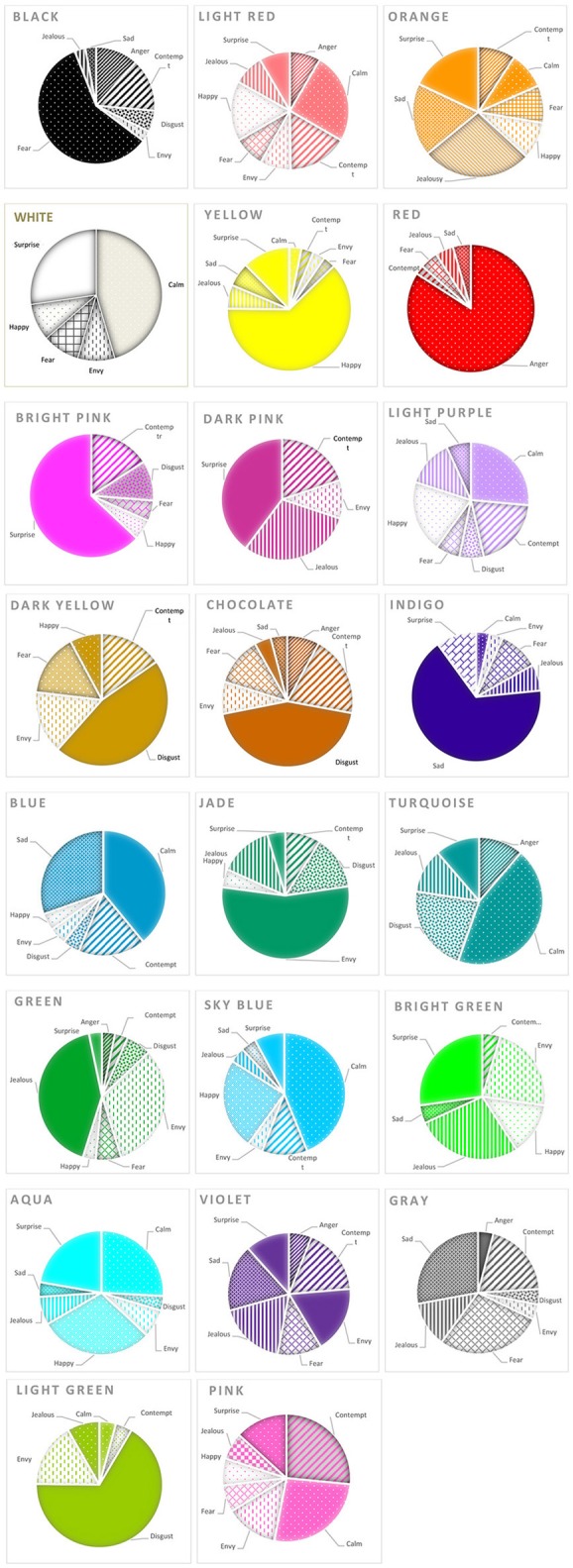
Frequency of selection of emotions for each color: study 1.

“Red” was indicated among the top three colors for *anger*, followed by *jealousy, fear*, and *envy*, respectively (Figure 2). The intensity of “red” for *anger* was high, whereas the intensity for “red” given to the other emotions was low-moderate (Table 2). The intensities of “red” among the emotions differed, χ${}_{\left(3,\;\text{N}=73\right)}^{2}$ = 99.22, *p* < 0.001, as did the intensities of “red” between *anger* and *jealousy, p* < 0.001. The agreement among raters was relatively high, Kendall's *W* = 0.680. While these analyses suggest that “red” might be specific to *anger*, resampling the data showed that the probability of replicating this finding was well below the level of statistical acceptance. The probability of selecting a sample in which the intensity of “red” to *anger* exceeded that given to *fear* was around 62% (CI = −4:9). All other probabilities of selecting a sample in which the intensity of “red” was greater for one emotion compared to that of another emotion were < 44%. Therefore, despite frequency and intensity analyses which showed that the intensity of “red” assigned to *anger* differed from *jealousy*, re-sampling the same data suggested that we would expect to find this effect only about 62% of the time.

#### “Gray”

“Gray” was indicated among the top three colors for *sadness, fear*, and *contempt* (Figure 2). The intensity of “gray” for *sadness* was moderate, whereas the intensity of “gray” given to the other emotions was low (Table 2). The intensities of “gray” among the emotions differed, χ${}_{\left(3,\;\text{N}=73\right)}^{2}$ = 26.95, *p* < 0.001, as did the intensities between *sadness* and *fear, p* = 0.01. The agreement among raters was low, however, Kendall's *W* = 0.185. When we resampled our data, the probability of selecting a sample in which the intensity of “gray” to *sadness* exceeded that given to *fear* was about 52% (CI = −6:8). All other probabilities of selecting a sample in which the intensity of “gray” was greater for one emotion compared to another were < 46%. Therefore, we conclude the likelihood that “gray” is specific to *sadness* is quite low.

#### “Yellow”

“Yellow” was indicated among the top three colors for only two emotions, *happiness* and *surprise* (Figure 2). The intensity of “yellow” was high for *happiness*, and moderate for *surprise* (Table 2). The intensity of “yellow” given to *happiness* vs. that given to *surprise* differed, *p* < 0.05. The agreement among raters was low-moderate, however, Kendall *W* = 0.238. Using bootstrapping, we found that the probability of selecting a sample in which the intensity of “yellow” given to *happiness* exceeded that to *surprise* was about 63% (CI = −3:6). Therefore, the likelihood that yellow is specific to *happiness* is quite low.

#### “Green”

“Green” was indicated among the top three colors for two emotions, *envy* and *jealousy* (Figure 2). The intensity of “green” to *envy* and *jealousy* were both moderate (Table 2). The intensities of “green” between *envy* and *jealousy* differed, *p* < 0.05. The agreement among raters was low, however, Kendall's *W* = 0.148. Again, when we resampled our data, we found that the probability of selecting a sample in which the intensity of “green” given to *envy* exceeded that for *jealousy* was only about 25% (CI = −6:6). Therefore, we conclude that it is unlikely that “green” is specific to *envy*.

#### “Indigo”

“Indigo” was indicated among the top three colors for only one emotion, *sadness*, making it potentially specific (Figure 2). The intensity of “indigo” was moderate for *sadness* (Table 2), and the agreement among raters was low-medium, Kendall's *W* = 0.243. Using bootstrapping, we found that the probability of selecting a sample in which the intensity of “indigo” given to *sadness* exceeded the intensity of “indigo” to another emotion was always < 62%. Therefore, it is unlikely that “blue” is specific to *sadness*, even though participants never picked “indigo” as one of the top three colors for any other emotion.

#### “Bright Pink”

“Bright pink” was indicated among the top three colors for only one emotion, *surprise*, which suggested it might be specific to it (Figure 2). The intensity of “bright pink” was moderate to *surprise* (Table 2), yet the agreement among raters was also low, Kendall's *W* = 0.103. Using bootstrapping, we found that the probability of selecting a sample in which the intensity of “bright pink” to *surprise* exceeded that to another emotion was always < 63%. Therefore, it is unlikely that “bright pink” is specific to *surprise*, even though participants never picked “bright pink” as one of the top three colors for any other emotion.

#### “Chocolate,” “Dark Yellow,” and “Light Green”

“Chocolate,” “dark yellow,” and “light green” were only indicated among the top three colors for one emotion, *disgust* (Figure 2). This might suggest that these three colors are specific to this emotion. Yet, when we looked at the intensities of each color given to *disgust*, they were all moderate (Table 2), and the agreement among individuals for each color ranged from low to moderate, Kendall's *W* = 0.227, 0.285, 0.076, respectively.

Using bootstrapping, we found that the probability of selecting a sample in which the intensity of “chocolate” to *disgust* exceeded that to another emotion was always < 58%. Likewise, the probability of selecting a sample in which the intensity of “dark yellow” to *disgust* exceeded that to another emotion was always < 55%. Finally, the probability of selecting a sample in which the intensity of “light green” exceeded that for any other emotion was always < 59%. Therefore, none of these colors are likely to be specific to *disgust*, despite participants only picking these three colors as the top colors for *disgust*.

#### Summary Specificity

Nine of the 23 colors showed some evidence of specificity, yet agreement among raters in all conditions were low to moderate. The exception was “red” with *anger*, in which the agreement was high. Yet, when we resampled our data, even “red” as specific to *anger* was not likely to replicate. Study 2 shows that this is the case, as only one of the specificity pairings replicated.

It is also worth noting that when we analyzed the frequencies from the “forced choice” question (see Supplemental Materials), we found statistical evidence for six colors listed above. More specifically, we did not replicate the effects of “chocolate” and “gray,” yet we did find specificity for “jade” and “black” which we had not before. This was likely because “jade” was now specific to *envy* (“green,” which was also high for *envy*, was now specific to *jealousy*), and “black” was now specific to *fear* (“red,” which was also high for *fear*, was now specific to *anger*). Finally, “bright pink” was specific, but now with *calmness* (not *surprise*)! These results show how restraining the choice of colors (to one color vs. several colors) can profoundly affect the pairings that emerge, suggesting that not only are individual color-emotion pairings likely specific to one experiment, but also to how (within the same experiment) the question is asked.

### Consistency: Study 2

We used the same analyses as in study 1 for consistency. We did not bootstrap our data in this study since the primary purposes was to show that effects which do emerge within the context of an experiment are typically experiment-specific, and not likely to replicate in another. We report analyses for consistency which did not reach statistical significance in Supplemental Materials. Alpha for all analyses was set to 0.05.

#### Disappointment

**Figure 3:**
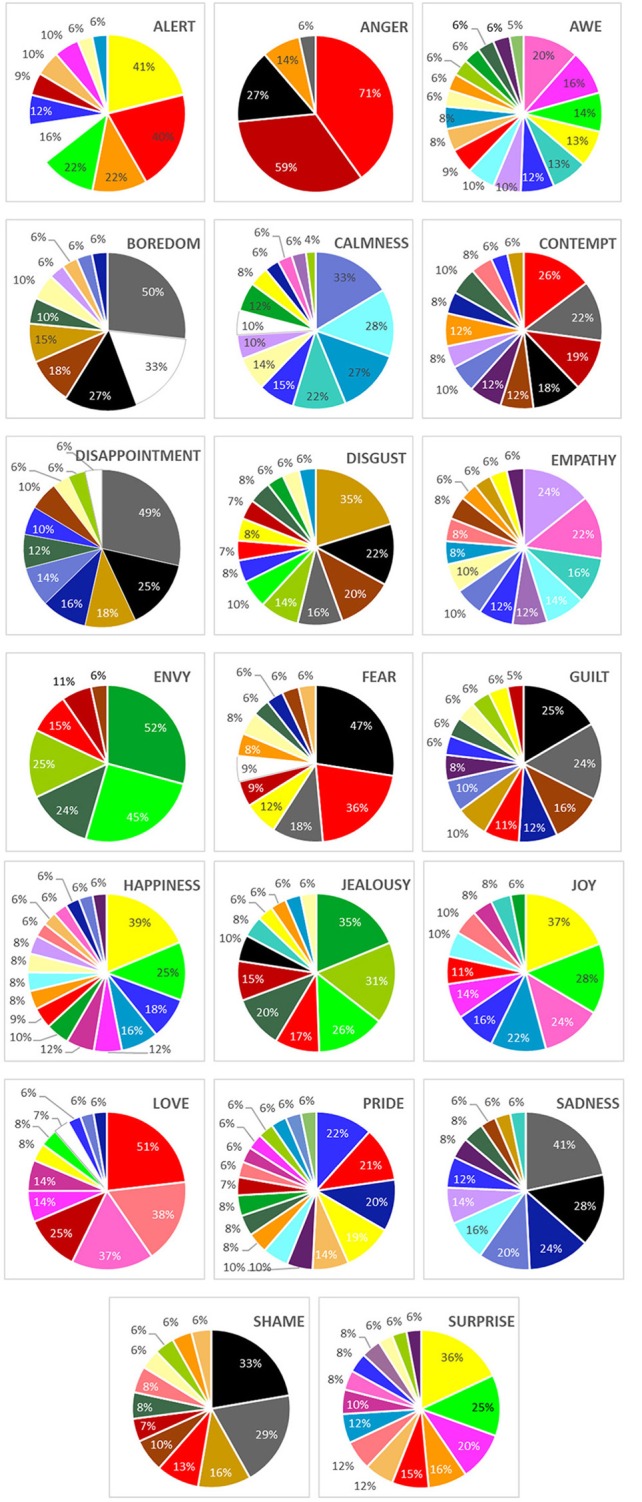
Frequency of selection of colors for emotion: study 2. Colors represent the actual color swatches. See Appendix A to associate the color name with each color. Colors with < 5% frequency rating are not depicted in pie charts.

“Gray” was the top-ranked color for the emotion *disappointment*, followed by “black” and “dark yellow,” respectively (Figure 3). Overall, participants assigned different intensities to the top three-ranked colors, χ${}_{\left(2,\;N=52\right)}^{2}$ = 16.00, *p* < 0.001. In addition, the intensities assigned to “gray” statistically differed from intensities assigned to “black,” *p* = 0.05, yet there was low agreement among individuals, Kendall's *W* = 0.154 (Table 3).

**Table 3:** Raw mean intensity (followed by SD) of the top colors selected for study 2.

**Coloremotion**	**“Black”**	**“Red”**	**“Gray”**	**“Yellow”**	**“Light Purple”**	**“Dark Red”**	**“Orange”**	**“Green”**	**“Brown”**	**“Dark Blue”**	**“Blue”**	**“Bright Pink”**	**“Bright Green”**	**“Dark Yellow”**	**“Pink”**	**“White”**	**“Periwinkle”**	**“Light Blue”**	**“Dark Green”**	**“Bright Blue”**	**Light Red”**	**“Light Green”**
Alert	X	3.5 4.4	X	3.6 4.43	X	X	1.8 3.6	X	X	X	X	X	X	X	X	X	X	X	X	X	X	**X**
Anger	2.1 3.7	6.4 4.3	X	X	X	5.2 4.5	X	X	X	X	X	X	X	X	X	X	X	X	X	X	X	X
Awe	X	X	X	X	X	X	X	X	X	X	X	1.2 3.1	1.1 2.9	X	1.4 3.1	X	X	X	X	X	X	X
Boredom	2.1 3.6	X	4.3 4.5	X	X	X	X	X	X	X	X	X	X	X	X	2.7 4	X	X	X	X	X	X
Calmness	X	X	X	X	X	X	X	X	X	X	2.1 3.6	X	X	X	X	X	2.9 4.2	2.1 3.6	X	X	X	X
Contempt	X	2.2 3.7	1.6 3.2	X	X	1.5 3.4	X	X	X	X	X	X	X	X	X	X	X	X	X	X	X	X
Disappointment	2.5 3.9	X	4 4.4	X	X	X	X	X	X	X	X	X	X	1.3 2.9	X	X	X	X	X	X	X	X
Disgust	1.6 3.2	X	X	X	X	X	X	X	1.5 3.4	X	X	X	X	2.2 3.7	X	X	X	X	X	X	X	X
Empathy	X	X	X	X	1.8 3.4	X	X	X	X	X	X	X	X	X	1.7 3.3	X	X	1.1 2.8	X	X	X	X
Envy	X	X	X	X	X	X	X	4.5 4.5	X	X	X	X	3.4 4.1	X	X	X	X	X	2.1 3.9	X	X	X
Fear	4.1 4.6	2.2 3.8	1.6 3.5	X	X	X	X	X	X	X	X	X	X	X	X	X	X	X	X	X	X	X
Guilt	2.3 4.1	X	2 3.6	X	X	X	X	X	1.1 2.8	X	X	X	X	X	X	X	X	X	X	X	X	X
Happy	X	X	X	3.5 4.6	X	X	X	X	X	X	X	X	2 3.5	X	X	X	X	X	X	1.4 3.2	X	X
Jealousy	X	X	X	X	X	X	X	2.8 4	X	X	X	X	2.1 3.7	X	X	X	X	X	X	X	X	2.3 3.5
Joy	X	X	X	3.1 4.3	X	X	X	X	X	X	X	X	2.1 3.7	X	1.9 3.7	X	X	X	X	X	X	X
Love	X	4.8 4.8	X	X	X	X	X	X	X	X	X	X	X	X	3 4.1	X	X	X	X	X	3.1 4.3	X
Pride	X	1.7 3.5	X	X	X	X	X	X	X	1.6 3.5	X	X	X	X	X	X	X	X	X	1.8 3.6	X	X
Sad	2.4 4.1	X	3.5 4.3	X	X	X	X	X	X	1.7 3.4	X	X	X	X	X	X	X	X	X	X	X	X
Shame	2.9 4.3	X	2.4 3.8	X	X	X	X	X	X	X	X	X	X	1.2 2.9	X	X	X	X	X	X	X	X
Surprise	X	X	X	3.1 4.2	X	X	X	X	X	X	X	1.6 3.4	2 3.6	X	X	X	X	X	X	X	X	X

*Only colors listed which were among the top three for any emotion are listed. X indicates not compared because color was not among the top three by frequency for that emotion*.

#### Love

“Red” was the top-ranked color for the emotion *love*, followed by “light red” and “pink,” respectively (Figure 3). Overall, participants gave different intensities to the top three-ranked colors, χ${}_{\left(2,\;N=52\right)}^{2}$ = 11.06, *p* = 0.01. In addition, the intensities assigned to “red” were statistically different from “light red,” *p* = 0.04, yet there was low agreement among individuals, Kendall's *W* = 0.106 (Table 3).

#### Summary Consistency

Only *love* and “red,” and *disappointment* and “gray” were consistent pairings in study 2. Neither of these emotions were included in study 1. Of the four emotions for which we found consistent pairings in study 1 (*anger, envy, fear, happy*), none of these replicated, although the top-indicated color (e.g., “red,” “green,” “black,” and “yellow,” respectively) were the same top-indicated colors in this study for these emotions.

To address the concerns that participants perceived the colors differently since they could use their own device (despite our efforts to limit the survey to users of the iPhone and instruct participants to adjust their screen brightness), we compared the top-indicated color for each emotion from study 2 to the lab control. For 13 of the 20 emotions, participants indicated the same top color for both studies. For the remaining seven emotions, what was the top color for study 2 was either second or third (or vice versa) in the lab control. We interpret this to mean that participants did *not* perceive the colors on their devices differently despite their ability to complete the survey in any lightning conditions. The amount of small variability we found was still qualitatively less than between the main results of studies 1 and 2.

### Specificity: Study 2

We used the same analyses as in study 1 for specificity. Again, we did not bootstrap the data. As in study 1, we analyzed each color separately. We report colors that did not meet statistical significance in Supplemental Materials.

#### “Dark Red”

**Figure 4:**
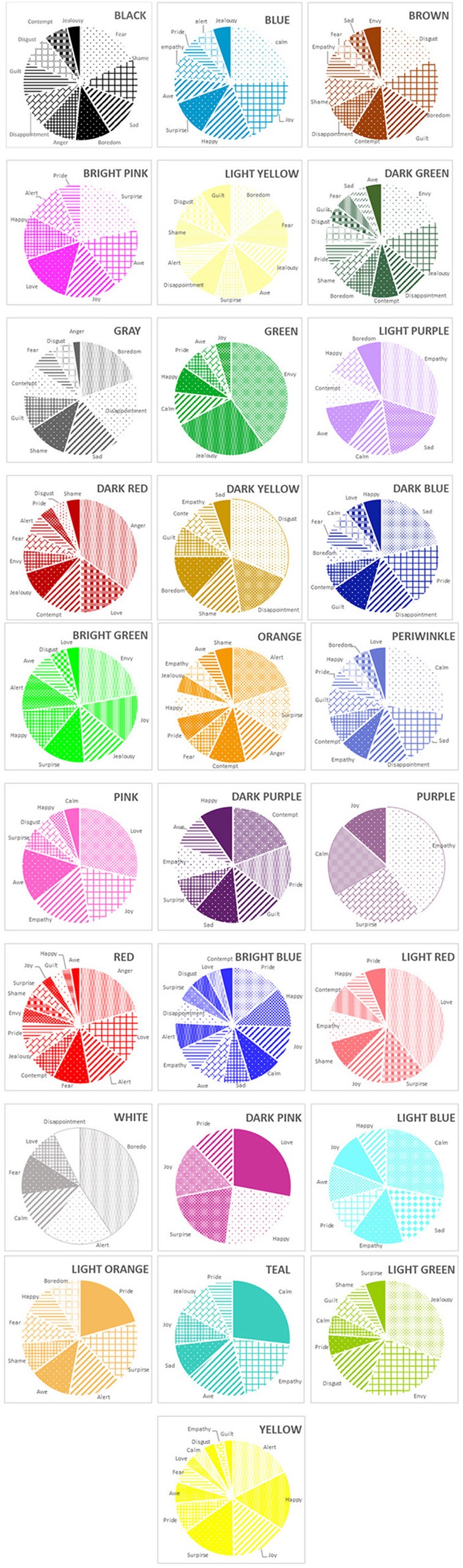
Frequency of selection of emotions for each color: study 2.

*Anger* was the top ranked emotion for the color “dark red,” followed by *love*, and *contempt*, respectively (Figure 4). Overall, participants assigned different intensities to the top three-ranked emotions, χ${}_{\left(2,\;N=52\right)}^{2}$ = 18.37, *p* < 0.001, and the intensities assigned to *anger* statistically differed from intensities assigned to *love, p* = 0.01. Overall, however, agreement among individuals was low, Kendall's *W* = 0.177 (Table 3).

#### “Green”

*Envy* was the top ranked emotion for the color “green,” followed by *jealousy*, and *calmness*, respectively (Figure 4). Overall, participants assigned different “green” color intensities to the top three-ranked emotions, χ${}_{\left(2,\;N=52\right)}^{2}$ = 23.15, *p* < 0.001. In addition, intensities associated with *envy* significantly differed from intensities associated with *jealousy, p* = 0.04, and agreement was moderate, Kendall's *W* = 0.223 (Table 3).

#### “Light Red”

*Love* was the top ranked emotion for the color “light red,” followed by *surprise* and *joy*, respectively (Figure 4). Participants gave different intensities to the top three-ranked emotions, χ${}_{\left(2,\;N=52\right)}^{2}$ = 15.39, *p* < 0.001. In addition, intensities assigned to *love* statistically differed from intensities assigned to *surprise, p* = 0.04, yet the agreement among individuals was still low, Kendall's *W* = 0.148 (Table 3).

#### Summary Specificity

Three of the 28 colors showed statistical specificity: “dark red” was specific to *anger*, “green” with *envy*, and “light red” with *love*. Nonetheless, agreement among raters in all conditions was low to moderate. Of the specific pairings in study 1, only “green” with *envy* replicated.

### HSL Dimensions: Study 2

Finally, to test our hypothesis (Hypothesis 2) that the individual facets of color (HSL) would better predict the color-emotion pairings than the perceived color (referred to by the color name), we ran a series of nominal (categorical) regressions. First, we ran a categorical regression using HSL combined as predictors with emotion as the target. Then we ran the same regression with color (referred to by color name) as the predictor. Finally, we looked at how the model changed with HSL added in individually. This allowed us to get a sense of which facet predicted the most agreement. From this we could compare with previous studies that suggested lightness and saturation are mainly responsible for emotion-color pairings.

We found that HSL together produced a significant model, *F*_(5, 50)_ = 52.15, *p* < 0.001, *r*^2^ = 0.84 (standardized B = −0.80, *p* = 0.008, hue; standardized B = −0.21, *p* = 0.080, saturation; standardized B = −0.15, *p* = 0.145, lightness). Although the model was significant, only hue was a significant predictor. This was confirmed when we tested each predictor separately: The model was significant with hue, *F*_(1, 54)_ = 156.56, *p* < 0.001, *r*^2^ = 0.74, but also with only saturation, *F*_(1, 80)_ = 14.38, *p* < 0.001, *r*^2^ = 0.15. Lightness remained non-significant, *p* = 0.080. Color (as named) did not produce a significant model, *F*_(2, 81)_ = 2.06, *p* = 0.134, *r*^2^ = 0.05. These findings confirm our second hypothesis that individual facets of color, namely hue and saturation, predicted agreement of emotion assignment better than the perceived color. Unlike other previous studies (e.g., Valdez and Mehrabian, 1994; Gao et al., 2007), however, lightness did not have an effect, whereas hue did have an effect.

## Discussion

An evolutionary approach to emotion is consistent with the idea that there should be specificity and consistency for color-emotion pairings. Consistency suggests that an emotion is reliably paired with a color(s). Specificity suggests that a color(s) is specific to an emotion.

We performed two studies, in which English-speaking participants completed a similar online survey assessing their color-emotion pairing by selecting up to three colors for each emotion and indicating an intensity. In study 2, we added more emotions and colors. We also conducted a laboratory control study in which new participants completed the survey under controlled lighting conditions and the color on the computer monitor was calibrated for each participant. Study 1 had the additional unique question of asking participants to pick one, different emotion for each emotion, in a “forced choice” format which is more similar to the previous research on this topic.

In study 1, we found that only four emotions showed any evidence of color consistency, and only nine colors showed any evidence of emotion specificity. When we resampled our data, however, there was no evidence that any of these effects would be retained. Bootstrapping revealed no evidence that this would replicate with statistical certainty, consistent with our hypothesis that individual color-emotion pairings are due to the experimental context and not universal or stable across participants and formats. Interestingly, and perhaps more telling, is that when participants in study 1 were forced to choose only one color, “green” was now specific to *jealousy*, emphasizing the instability of color-emotion pairings when only the formatting of the question changes.

It is important to note that bootstrapping, which is now considered a “gold standard” (if not a necessity) in data collection, provides the ability to draw hundreds or thousands of samples from the sample distribution rather than relying on only the sample distribution. To this end, the standard error from resampling is a much better estimate of the standard deviation. The results are comparable to performing the experiment hundreds of thousands of times on the same participants.

In study 2, we found only two emotions that showed any evidence of color consistency, and only three colors that showed any evidence of emotion specificity. In addition, the individual pairings of consistency and specificity we found for study 1 were different from those in study 2, with the exception that “green” was specific to *envy* in both studies. We also confirmed that participants' judgments are not influenced by perceiving the colors differently based on the device on which they take the survey, since the top-indicated color was the same across the majority of emotions between the laboratory control study and the results reported herein.

Without strong evidence for consistency and specificity for emotion-color pairings, why does it feel as if *anger is* “red” and that *envy is* “green”? One reason is that previous investigations often severely limit the range of answer choices which imposes consistency (and erroneously is used as an indication of the existence of a diagnostic signal) (for more on this, see Barrett, 2006a,b). Said another way, the context created by the study can inflate agreement. Therefore, when like-minded people are asked to associate emotions with colors from a restrictive set, they are likely to use the provided answer choices and verbal labels to make “best guesses.”

Another reason is that our folk psychology—that which comes from our culture and is reinforced by our culture (i.e., popular movies and books which continue to portray such pairings)—reinforces these relationships. For example, in many American films, “good guys” are often dressed in white and “bad guys” in black (Frank and Gilovich, 1988; Meier et al., 2004). In Hellenistic, Roman, and Christian traditions, “white” is the color of joy, innocence, and purity, whereas “black” is the color associated with evil (e.g., “Satan is the Prince of Darkness” and “Jesus is the Light of the World”) (Meier et al., 2007; Chiou and Cheng, 2013; see Meier et al., 2004). Allah is equated with light in the Koran, and truth is characterized as a “light” or “lamp” in Buddhist writings (Meier et al., 2004). Metaphors not only communicate abstract concepts, but they might also be necessary for grounding them as well (Gibbs, 1992; Lakoff and Johnson, 1999; Lakens et al., 2012, 2013). This is true of emotion, too (e.g., Barrett et al., 2007). In English, we have sayings such as: “hot-headed” or “red in the face” to refer to *anger*, “feeling blue” to refer to *sadness*, and “the green-eyed monster” to refer to *envy*.

According to The Theory of Constructed Emotion (Barrett, 2017), as a child learns more emotion words, s(he) becomes better at perceiving emotion and shows more granular categories. In this view, emotion is no different from other abstract categories which are learned during development. Emotion words help to create discrete emotion categories because they help to activate situated conceptualizations which might increase the processing of sensory information that is consistent with such conceptualizations (Barrett, 2006a,b; Wilson-Mendenhall et al., 2011; Lindquist and Gendron, 2013; see Lupyan and Ward, 2013; Fugate and Barrett, 2014; Lindquist et al., 2015).

We believe this to be similar for color language. For example, when people are asked to think about color-emotion pairings, they reference language. For instance, a person might reference linguistic phrases and metaphors that help clarify *what color anger should be* (e.g., “red in the face” or “seeing red”, Fetterman et al., 2012). They might also access the learned relationships between “red” and warning signals (e.g., fire alarms, stop signs, red ink), which highlight the need for vigilance and avoidance which are shared aspects with “anger” (see Elliot et al., 2007). Admittedly, the purpose of this paper was not to test the reasons behind any potential pairings, as much as it was to see whether such pairings exist and whether colors are specific to emotions. Future research should indeed further explore the reasons behind beliefs in color-emotion associations as well as explore differences in color-emotion agreement cross-culturally.

Although study 2 addressed several limitations of study 1, there remain several possible influences on color-emotion pairings which we could not or did not control. For example, even though we included more colors and more emotions in study 2, there are over 1,800 notations listed in the Munsell book of colors. We also included emotions which have been referenced in the psychological literature as “potentially basic”: We did not use emotional adjectives as some previous studies have done.

While there are some shortcomings to this research, we believe that the strengths lie in deriving our hypotheses with respect to emotion theory which we statistically test. In addition, we present our colors as swatches rather than color words (paying attention to HSL), and allow participants to indicate a strength of agreement for up to three colors for each emotion. We also compare people's agreement using this format with a more traditional format in which participants were asked to choose one color for each emotion. Finally, we bootstrapped our data in study 1 to show that the likelihood of replicating any individual color-emotion pairing was well-below the accepted level of statistical significance.

## Author Contributions

JF was involved in all aspects of the research. CF helped disseminate the survey, reviewed the literature, prepared data for analysis, analyzed data, and aided in figure preparation. She was also responsible for most aspects of study 2. Both authors approved the final revised manuscript for submission. The authors declare no conflicts of interest. Portions of this data were presented at the Psychonomics conference in 2015 and at the American Psychological Association conference in 2018.

### Conflict of Interest Statement

The authors declare that the research was conducted in the absence of any commercial or financial relationships that could be construed as a potential conflict of interest.

## Footnotes

^1^The one exception was a theoretical model designed by Plutchik (1980) in which he arranged emotions around a color wheel, which was motivated by the analogy between the opponent–color theory and the circularity of emotions (cf. Hanada, 2017, p. 3–4). He proposed that there are eight primary emotions consisting of four pairs of opposite emotions (joy–sadness, trust–disgust, fear–anger, surprise–anticipate) like complementary colors. Other emotions are mixtures of primary emotions, like other colors are mixtures of primary colors. Although he implied similarities between the color and emotion circle, he did not seem to argue that his emotion circle matched the color circle (cf. Hanada, 2017, p. 3–4). Interestingly, although commonly referred to in the media and popular psychology, there is little to no evidence that his color-emotion pairings were ever empirically tested or supported.

^2^The role that language has on emotion perception has grown within the last 10 years, and many studies now show that words matter to how perceivers not only categorize but also perceive the emotional world (For reviews, see Fugate, 2013; Lindquist and Gendron, 2013; Lindquist et al., 2015; see Fugate et al., 2018). Accordingly, color-related terms, including basic colors within a language, are likely to play a role on how we both perceive and link color with emotion. For example, English has many color-emotion phrases which might influence color-emotion pairings (e.g., “red with *rage*,” “green with *envy*,” “feeling blue”).

^3^The survey was taken on a Dell Latitude E5570 (5th Generation Intel Core i5 processor, 16 GB of RAM, a 256GB SSD hard drive, with a 1920 × 1080 (15inch) screen display). A pantone huey (MEU101) monitor calibration device (colorimeter) was used to calibrate the computer screen prior to each participant. All participants completed the survey in a dark room.

^4^The standard parametric approach to hypothesis testing can only quantify whether the mean intensity rating for one color is significantly greater than the mean intensity rating for another color. Instead, we used a non-parametric bootstrapping method to more precisely quantify the probability that a given intensity rating for “red” is significantly greater than a given intensity rating for “black”. We first sampled with replacement 10,000 samples from the intensity ratings for “red” and another 10,000 samples from the intensity rating for “black”. We then computed 10,000 difference pairs by subtracting away the intensity for “black” from that of “red”. The fraction of the difference pairs greater than zero is the desired probability. In this case, probabilities smaller than 0.95 are not significant.

^5^Our confidence intervals are based on intensity ratings, which ranged between 1 and 10.
