# Supplementary material for: What Color Is Your Anger? Assessing Color-Emotion Pairings in English Speakers
Source: Front Psychol. 2019 Feb 26;10:206. doi: 10.3389/fpsyg.2019.00206 (PMC6399154; doi:10.3389/fpsyg.2019.00206)
Supplement: Supplementary file 1 [file Data_Sheet_1.pdf]

Supplementary Materials

I. Frequency Analysis for Forced Choice Question: Study 1

Consistency. Sixty-two people contributed to the first set of analyses. Since participants' selections for this question were nominal/categorical, we used nonparametric frequency analyses. For each emotion, we ran Cochran's Q tests to compare whether the frequencies of colors differed from each other. Emotions appear in alphabetical order. Bolded emotions represent statistical significance.

Anger. "Red" was the top-ranked color associated with the emotion *anger*, followed by "black" and "gray", respectively. Overall, participants assigned different frequencies to the top three-ranked colors, $\chi 2(2) = 64.75$, $p < .001$; in addition, "red" frequencies significantly differed from "black" frequencies, $p < .001$.

Calm. "Blue" was the top-ranked color associated with the emotion *calm*, followed by "sky blue" and "aqua", respectively. Overall, frequencies did not differ between the top three-ranked colors, $\chi 2(2) = 1.27$, $p = .53$.

Contempt. "Chocolate" was the top-ranked color associated with the emotion *contempt*, followed by "black" and "gray", respectively. Overall, frequencies did not differ between the top three-ranked colors, $\chi 2(2) = 0.33$, $p = .85$.

Disgust. "Light green" was the top-ranked color associated with the emotion *disgust*, followed by "dark yellow" and "chocolate", respectively. Overall, frequencies did not differ between the top three-ranked colors, $\chi 2(2) = 1.00$, $p = .61$.

Envy. "Green" was the top-ranked color associated with the emotion *envy*, followed by "jade" and "bright green", respectively. Overall, frequencies did not differ between the top three-ranked colors, $\chi 2(2) = 1.77$, $p = .41$.

<u>Fear</u>. “Black” was the top-ranked color associated with the emotion *fear*, followed by “gray” and “chocolate”, respectively. Overall, participants assigned different frequencies to the top three-ranked colors, $\chi 2(2) = 23.46$, $p < .001$; in addition, “black” frequencies significantly differed from “gray” frequencies, $p < .001$.

<u>Happiness.</u> “Yellow” was the top-ranked color associated with the emotion *happiness*, followed by “aqua” and “sky blue”, respectively. Overall, participants assigned different frequencies to the top three-ranked colors, $\chi 2(2) = 15.12$, $p < .001$; in addition, “yellow” frequencies significantly differed from “aqua” frequencies, $p = .02$.

<u>Jealousy.</u> “Green” was the top-ranked color associated with the emotion *jealousy*, followed by “bright green” and a three-way tie between “jade” “orange”, and “dark pink”, respectively. Overall, participants assigned different frequencies to the top three-ranked colors, $\chi 2(2) = 15.43$, $p < .004$; however, “green” frequencies did not significantly differ from “bright green” frequencies, $p = .10$.

<u>Sadness.</u> “Indigo” was the top-ranked color associated with the emotion *sad*, followed by “gray” and “blue”, respectively. Overall, participants assigned different frequencies to the top three-ranked colors, $\chi 2(2) = 6.73$, $p = .04$; however, “indigo” frequencies did not significantly differ from “gray” frequencies, $p = .14$.

<u>Surprise.</u> “Bright pink” was the top-ranked color associated with the emotion *surprise*, followed by “yellow” and “aqua”, respectively. Overall, frequencies did not differ between the top three-ranked colors, $\chi 2(2) = 3.59$, $p = .17$.

Specificity. We applied the same logic as above, but for each color. For each color, we ran Cochran’s Q tests to compare whether the frequencies of emotions differed from each other. Colors appear in alphabetical order. Colors appear in alphabetical order. Bolded colors represent statistical significance.

Aqua. *Happiness* was the top-ranked emotion associated with the color "aqua", followed by *calmness* and *jealousy*, respectively. Overall, frequencies did not differ between the top three-ranked emotions, $\chi 2(2) = 1.08$, $p = .58$.

Black. *Fear* was the top-ranked emotion associated with the color "black", followed by a tie between *anger* and *contempt,* and then by *disgust*, respectively. Overall, participants assigned different frequencies to the top three-ranked colors, $\chi 2(2) = 42.03$, $p < .001$; in addition, *fear* frequencies significantly differed from both *anger* and *contempt* frequencies, $p < .001$.

Blue. *Calmness* was the top-ranked emotion associated with the color "blue", followed by *sadness* and *contempt*, respectively. Overall, frequencies did not differ between the top three-ranked emotions, $\chi 2(2) = 4.88$, $p = .09$.

Bright Green. *Envy* was the top-ranked emotion associated with the color "bright green", followed by *jealousy* and *surprise*, respectively. Overall, frequencies did not differ between the top three-ranked emotions, $\chi 2(2) = 0.29$, $p = .87$.

Bright Pink. *Calmness* was the top-ranked emotion associated with the color "bright pink", followed by *contempt,* and *happiness*, respectively. Overall, participants assigned different frequencies to the top three-ranked colors, $\chi 2(2) = 17.43$, $p < .001$; in addition, *calmness* frequencies significantly differed from *contempt* frequencies, $p < .001$.

Chocolate. *Disgust* was the top-ranked emotion associated with the color "chocolate", followed by *contempt* and *fear*, respectively. Overall, frequencies did not differ between the top three-ranked emotions, $\chi 2(2) = 4.26$, $p = .12$.

Dark Pink. *Surprise* was the top-ranked emotion associated with the color "dark pink", followed by *contempt* and *fear*, respectively. Overall, frequencies did not differ between the top three-ranked emotions, $\chi 2(2) = 4.26$, $p = .12$.

Dark Yellow. *Disgust* was the top-ranked emotion associated with the color "dark yellow", followed by *contempt,* and *envy*, respectively. Overall, participants assigned different frequencies to the top three-ranked colors, $\chi 2(2) = 9.70$, $p = .01$; in addition, *disgust* frequencies significantly differed from *contempt* frequencies, $p = .03$.

Gray. *Sadness* was the top-ranked emotion associated with the color "gray", followed by *fear* and *contempt*, respectively. Overall, frequencies did not differ between the top three-ranked emotions, $\chi 2(2) = 4.69$, $p = .10$.

Green. *Jealousy* was the top-ranked emotion associated with the color "green", followed by *envy,* and *disgust*, respectively. Overall, participants assigned different frequencies to the top three-ranked colors, $\chi 2(2) = 10.75$, $p = .01$; however, *jealousy* frequencies did not significantly differ from both *envy* frequencies, $p = .67$.

Indigo. *Sadness* was the top-ranked emotion associated with the color "indigo", followed by *surprise* and *fear*, respectively. Overall, participants assigned different frequencies to the top three-ranked colors, $\chi 2(2) = 28.22$, $p < .001$; in addition, *sadness* frequencies significantly differed from *surprise* frequencies, $p < .001$.

Jade. *Envy* was the top-ranked emotion associated with the color "jade", followed by *jealousy* and *disgust*, respectively. Overall, participants assigned different frequencies to the top three-ranked colors, $\chi 2(2) = 9.10$, $p = .01$; in addition, *envy* frequencies significantly differed from *jealousy* frequencies, $p = .01$.

Light Green. *Disgust* was the top-ranked emotion associated with the color "light green", followed by *envy* and *contempt*, respectively. Overall, participants assigned different frequencies to the top three-ranked colors, $\chi 2(2) = 15.25$, $p < .001$; in addition, *disgust* frequencies significantly differed from *envy* frequencies, $p < .003$.

Light Purple. *Calmness* was the top-ranked emotion associated with the color "light purple", followed by *happiness* and *contempt*, respectively. Overall, frequencies did not differ between the top three-ranked emotions, $\chi 2(2) = 1.40$, $p = .49$.

Light Red. *Calmness* was the top-ranked emotion associated with the color "light red", followed by *happiness* and *envy*, respectively. Overall, frequencies did not differ between the top three-ranked emotions, $\chi 2(2) = 1.00$, $p = .61$.

Orange. *Jealousy* was the top-ranked emotion associated with the color "orange", followed by *contempt* and *surprise*, respectively. Overall, frequencies did not differ between the top three-ranked emotions, $\chi 2(2) = 1.00$, $p = .61$.

Pink. *Calmness* was the top-ranked emotion associated with the color "pink", followed by *contempt* and *envy*, respectively. Overall, frequencies did not differ between the top three-ranked emotions, $\chi 2(2) = 0.80$, $p = .67$.

Red. *Anger* was the top-ranked emotion associated with the color "red", followed by *fear* and *sadness*, respectively. Overall, participants assigned different frequencies to the top three-ranked colors, $\chi 2(2) = 28.22$, $p < .001$; in addition, *sadness* frequencies significantly differed from *surprise* frequencies, $p < .001$.

Sky Blue. *Calmness* was the top-ranked emotion associated with the color "sky blue", followed by *happiness* and *contempt*, respectively. Overall, frequencies did not differ between the top three-ranked emotions, $\chi 2(2) = 4.23$, $p = .09$.

Turquoise. *Calmness* was the top-ranked emotion associated with the color "turquoise", followed by *disgust* and *contempt*, respectively. Overall, frequencies did not differ between the top three-ranked emotions, $\chi 2(2) = 4.67$, $p = .10$.

Violet. *Envy* was the top-ranked emotion associated with the color "violet", followed by *contempt* and *jealousy*, respectively. Overall, frequencies did not differ between the top three-ranked emotions, $\chi 2(2) = 0.20$, $p = .91$.

White. *Calmness* was the top-ranked emotion associated with the color "white", followed by *surprise* and *fear*, respectively. Overall, frequencies did not differ between the top three-ranked emotions, $\chi 2(2) = 3.82$, $p = .15$.

Yellow. *Happiness* was the top-ranked emotion associated with the color "yellow", followed by *surprise* and *jealousy*, respectively. Overall, participants assigned different frequencies to the top three-ranked colors, $\chi 2(2) = 22.11$, $p < .001$; in addition, *happiness* frequencies significantly differed from *surprise* frequencies, $p = .003$.

II. Non Statistically Significant Results Reported for Study 1

Consistency. We did not resample our data for these non-significant effects.

Calmness. "Sky blue" was indicated as the most frequent color, followed by "blue" and then "light purple" (Figure 1). All three colors had moderate intensities (Table 1). The intensities among the three colors did not differ, χ^2 (2, N = 73), and neither was the difference between "sky blue" and "blue." Agreement was low, Kendall's $W = .007$. Therefore, we suggest that there is little evidence for strong consistency between *calmness* and any color(s).

Contempt. "Black" was most frequently chosen, followed by a tie between "light purple" and "gray" (Figure 1). In addition, the intensities among these three colors were all quite low (Table 1). The intensities among the three colors did not differ, χ^2 (2, N = 73) = 3.66, and neither was the difference between "black" and either "gray". Agreement was low, Kendall's $W= .025$. Therefore, we suggest that there is little evidence for strong consistency between *contempt* and any color(s).

Disgust. "Chocolate" was the most frequently indicated color, followed by "dark yellow" and then "light green" (Figure 1). All colors had moderate intensities (Table 1). The intensities among the three colors did not differ significantly, χ^2 (2, N = 73) = 0.93, and neither did "chocolate" and "dark yellow". Although it might be possible that all three colors work together or are separately associated with *disgust* (mainly since the combined frequency of all three was over 50%), the lack of agreement among raters suggests that is fairly unlikely (Kendall's W = .006)

Envy. "Green" was most frequently chosen color, followed by "jade" and then "red" (Figure 1). All three colors had moderate intensities (Table 1). The intensities among the three colors did not differ, χ^2 (2, N = 73) = 3.07, and neither did "green" and "jade". There was low agreement among raters, Kendall's W = .021.

Jealousy. "Red" was the most frequent color, followed by "jade" and "green" (Figure 1). All three colors' intensities were moderate (Table 1). The intensities among the three colors did not differ significantly, χ^2 (2, N = 73) = 1.65. Agreement was low, Kendall's W = .011.

Surprise. "Yellow" and "bright pink" were tied as the most frequently picked colors for *surprise*, followed by "aqua" (Figure 1). All three colors had moderate intensities (Table 1). The intensities among the three colors differed, χ^2 (2, N = 73) = 1.06, but obviously that between the top two color did not since they were identical. The agreement was low-moderate among participants, Kendall's W = .007.

Specificity. Because of the lack of statistical significance, we did not resample our data. Eight colors were not analyzed because they were selected infrequently (see Results).

"Black". *Fear* was the top ranked emotion, followed by *anger, sadness, and contempt,* respectively (Figure 2). The intensities given to *fear* and *anger* were high, whereas the intensities given to *sadness* and *contempt* were low-moderate (Table 1). The intensities among the four

emotions differed, χ^2 (3, N = 73) = 41.65, $p < .001$, yet that between *fear* and *anger* was not significant. Agreement was moderate, Kendall's W= .19.

"Light Purple". C*almness* was the top ranked emotion, followed by *contempt* and *surprise* (Figure 2). The intensity given to *calmness* was moderate, but low for *contempt* and *surprise* (Table 1). The intensities among the emotions differed, χ^2 (2, N = 73) = 7.66, $p < .05$, yet that between *calmness* and *contempt* did not. The agreement among raters was low, Kendall's W = .052.

"Sky Blue". *Calmness* was the top ranked emotion, followed by *happiness and surprise* (Figure 2). The intensities for *calmness* and *happiness* were moderate, but low for *surprise* (Table 1). The intensities among the emotions differed, χ^2 (2, N = 73) = 20.26, $p < .001$, but not between *calmness* and *happiness*. The agreement among raters was low, Kendall's W = .139.

"Jade". *Envy* was the top ranked emotion, followed by *jealousy* and *disgust* (Figure 2). The intensities for *envy* and *jealousy* were moderate, but low for *disgust* (Table 1). The intensities among the emotions differed, χ^2 (2, N = 73) = 15.44, $p < .001$, but not between *envy* and *jealousy*. The agreement among raters was low, Kendall's W = .106.

"Aqua". *Surprise* was the top ranked emotion, followed by *happiness* and *calmness* (Figure 2). All intensities were low to moderate (Table 1). The intensities among the emotions did not differ, χ^2 (2, N = 73). Agreement among raters was low, Kendall's W = .007.

"Blue". C*almness* was the top ranked emotion, followed by *sadness* and *happiness* (Figure 2). The intensities were moderate for *calmness* and *sadness*, but low for *happiness* (Table 1). Although the intensities of among the emotions differed, χ^2 (2, N = 73) = 9.75, p = .01, they did not between *calmness* and *sadness*. The agreement among raters was low, Kendall's W = .067.

III. Non Statistically Significant Results Reported for Study 2

Consistency.

Alert. "Red" was the top-ranked color associated with the emotion *alert*, followed by "yellow" and "orange", respectively. Overall, participants assigned different intensities to the top three-ranked colors, $\chi2(2) = 9.86$, $p = .01$; however, the intensities assigned to "red" did not statistically differ from intensities assigned to "yellow," $p = .96$, and the agreement among individuals was low. Kendall's $W = .095$.

Anger. "Red" was the top-ranked color associated with the emotion *anger*, followed by "dark red" and "black", respectively. Overall, participants assigned different intensities to the top three-ranked colors, $\chi2(2) = 24.31$, $p < .001$; however, the intensities assigned to "red" did not statistically differ from intensities assigned to "dark red," $p = .24$, with low-moderate agreement among individuals. Kendall's $W = .234$.

Awe. Among English speakers, "pink" was the top-ranked color associated with the emotion *awe*, followed by "bright pink" and "bright green", respectively. Overall, participants did not assign different intensities to the top three-ranked colors, $\chi2(2) = 0.67$, $p = .72$.

Boredom. "Gray" was the top-ranked color for the emotion *boredom*, followed by "white" and "black". Overall, participants assigned different intensities to the top three-ranked colors, $\chi2(2) = 14.31$, $p < .001$. However, the intensities assigned to "gray" were not statistically different from "white," $p = .13$, with low agreement among individuals. Kendall's $W = .138$.

Calm. "Periwinkle" was the top-ranked color for the emotion *calmness*, followed by "light blue" and "blue" ($M = 2.10$, $SD = 3.63$), respectively. Overall, participants did not assign different intensities to the top three-ranked colors, $\chi2(2) = 1.94$, $p = .38$. Kendall's $W = .019$.

Contempt. "Red" was the top-ranked color for the emotion *contempt,* followed by "gray" and "dark red", respectively. Overall, participants did not assign different intensities to the top three-ranked colors, $\chi2(2) = 0.88$, $p = .64$. Kendall's $W = .008$.

Disgust. "Dark yellow" was the top-ranked color for the emotion *disgust*, followed by "brown" and "light green", respectively. Overall, participants did not assign different intensities to the top three-ranked colors, $\chi2(2) = 4.15$, $p = .13$. Kendall'*s* $W = .063$.

Empathy. "Light purple" was the top-ranked color for the emotion *empathy*, followed by "pink" and "light blue", respectively. Overall, participants did not assign different intensities to the top three-ranked colors, $\chi2(2) = 1.82$, $p = .40$. Kendall's $W = .017$.

Envy. "Green" was the top-ranked color for the emotion *envy*, followed by "bright green" and "dark green", respectively. Overall, participants gave different intensities to the top three-ranked colors, $\chi2(2) = 14.22$, $p < .001$. However, the intensities assigned to "green" were not statistically different from "bright green," $p = .28$, and there was low agreement among individuals. Kendall's $W = .137$.

Fear. "Black" was the top-ranked color for the emotion *fear*, followed by "red" and "gray", respectively. Overall, participants gave different intensities to the top three-ranked colors, $\chi2(2) = 12.187$, $p < .001$. However, the intensities assigned to "black" were not statistically different from "red," $p = .09$, and there was with low agreement among individuals, Kendall's $W = .117$.

Guilt. "Black" was the top-ranked color for the emotion *guilt*, followed by "gray" and "brown", respectively. Overall, participants did not assign different intensities to the top three-ranked colors, $\chi2(2) = 5.22$, $p = .07$. Kendall's $W = .050$.

Happiness. "Yellow" was the top-ranked color for the emotion *happiness*, followed by "bright green", and "bright blue", respectively. Overall, participants assigned different intensities

to the top three-ranked colors, $\chi2(2) = 6.51$, $p = .04$. However, the intensities assigned to "yellow" did not differ from intensities assigned to "bright green", $p = .20$, and the agreement was low among individuals. Kendall's $W = .063$.

Jealousy. "Green" was the top-ranked color for the emotion *jealousy*, followed by "light green" and "bright green", respectively. Overall, participants did not assign different intensities to the top three-ranked colors, $\chi2(2) = 1.33$, $p = .52$. Kendall's $W = .013$.

Joy. "Yellow" was the top-ranked color for the emotion *joy*, followed by "bright green", and "pink", respectively. Overall, participants did not assign different intensities to the top three-ranked colors, $\chi2(2) = 4.81$, $p = .09$. Kendall's $W = .047$.

Pride. "Bright blue" ($M = 1.83$, $SD = 3.62$) was the top-ranked color for the emotion *pride*, followed by "red" ($M = 1.69$, $SD = 3.54$), and "dark blue" ($M = 1.63$, $SD = 3.45$), respectively. Overall, participants did not assign different intensities to the top three-ranked colors, $\chi2(2) = 0.12$, $p = .94$. Kendall's $W = .001$.

Sadness. "Gray" was the top-ranked color for the emotion *sadness*, followed by "black" and "dark blue", respectively. Overall, participants did not assign different intensities to the top three-ranked colors, $\chi2(2) = 5.85$, $p = .06$. Kendall's $W = .056$.

Shame. "Black" was the top-ranked color for the emotion *shame,* followed by "gray" and "dark yellow", respectively. Overall, participants assigned different intensities to the top three-ranked colors, $\chi2(2) = 7.43$, $p = .02$. However, the intensities assigned to "black" did not differ from those assigned to "gray", $p = .30$, with low agreement among individuals, Kendall's $W = .071$.

Surprise. "Yellow" was the top-ranked color for the emotion *surprise*, followed by "bright green" and "bright pink", respectively. Overall, participants did not assign different intensities to the top three-ranked colors, $\chi2(2) = 5.90$, $p = .06$. Kendall's $W = .057$.

Specificity.

"Black". *Fear* was the top ranked emotion for the color "black", followed by *shame*, and *anger*, respectively. Overall, participants assigned different intensities to the top three-ranked emotions, $\chi 2(2) = 8.72$, $p = .01$. However, the "black" color intensities assigned to fear did not statistically differ from the color intensities assigned to shame, $p = .43$, and the agreement was low. Kendall's $W = .084$.

"Blue". *Calmness* was the top ranked emotion for the color "blue", followed by *joy*, and *happiness*, respectively. Overall, participants did not assign different intensities to the top three-ranked emotions, $\chi 2(2) = 1.21$, $p = .55$. Kendall's $W = .012$.

"Bright Blue". *Pride* was the top ranked emotion for the color "bright blue", followed by *happiness*, and *joy*, respectively. Overall, participants did not assign different intensities to the top three-ranked emotions, $\chi 2(2) = 1.07$, $p = .59$. Kendall's $W = .010$.

"Bright Green". *Envy* was the top ranked emotion for the color "bright green", followed by *joy*, and *jealousy*, respectively. Overall, participants did not assign different intensities to the top three-ranked emotions, $\chi 2(2) = 4.72$, $p = .10$. Kendall's $W = .045$.

"Bright Pink". *Surprise* was the top ranked emotion for the color bright pink, followed by *awe*, and *love*, respectively. Overall, participants did not assign different intensities to the top three-ranked emotions, $\chi 2(2) = 1.61$, $p = .45$. Kendall's $W = .015$.

"Brown". *Disgust* was the top ranked emotion for the color "brown", followed by *boredom,* and *guilt*, respectively. Overall, participants did not assign different intensities to the top three-ranked emotions, $\chi 2(2) = 0.64$, $p = .73$. Kendall's $W = .006$.

"Dark Blue". *Sadness* was the top ranked emotion for the color "dark blue", followed by *pride* and *disappointment*, respectively. Overall, participants did not assign different intensities to the top three-ranked emotions, $\chi 2(2) = 0.63$, $p =.73$. Kendall's $W = .006$.

"Dark Green". *Envy* was the top ranked emotion for the color "dark green", followed by *jealousy*, and *disappointment*, respectively. Overall, participants did not assign different intensities to the top three-ranked emotions, $\chi 2(2) = 5.81$, $p = .06$. Kendall's $W = .056$

"Dark Pink". *Love* was the top ranked emotion for the color "dark pink", followed by *happiness,* and *surprise*, respectively. Overall, participants did not assign different intensities to the top three-ranked emotions, $\chi 2(2) = 0.29$, $p = .87$ Kendall's $W = .003$.

"Dark Purple". *Pride* was the top ranked emotion for the color "dark purple", followed by *sadness*, and *contempt*, respectively. Overall, participants did not assign different intensities to the top three-ranked emotions, $\chi 2(2) = 0.05$, $p = .98$. Kendall's $W = .000$.

"Dark Yellow". *Disgust* was the top ranked emotion for the color "dark yellow", followed by *disappointment*, and *shame*, respectively. Overall, participants assigned different intensities to the top three-ranked emotions, $\chi 2(2) = 10.64$, $p = .01$. The intensities assigned to *disgust*, however, did not statistically differ from intensities assigned to *disappointment*, $p = .11$, and agreement was low. Kendall's $W = .102$.

"Gray". *Boredom* was the top ranked emotion for the color "gray", followed by *disappointment*, and *sad,* respectively. Overall, participants did not assign different intensities to the top three-ranked emotions, $\chi 2(2) = 1.29$, $p = .86$. Kendall's $W = .003$.

"Light blue". *Calmness* was the top ranked emotion for the color "light blue", followed by *sadness*, and *empathy,* respectively. Overall, participants did not assign different intensities to the top three-ranked emotions, $\chi 2(2) = 3.01$, $p = .21$. Kendall's W = .030.

"Light Green". *Jealousy* was the top ranked emotion for the color "light green", followed by *envy,* and *disgust*, respectively. Overall, participants did not assign different intensities to the top three-ranked emotions, $\chi 2(2) = 5.41$, $p = .07$. Kendall's $W = .052$.

"Light Orange". *Pride* was the top ranked emotion for the color "light orange", followed by *alert*, and *shame,* respectively. Overall, participants did not assign different intensities to the top three-ranked emotions, $\chi 2(2) = 2.74$, $p = .26$. Kendall's $W = .026$.

"Light Purple". *Empathy* was the top ranked emotion for the color "light purple", followed by *sadness*, and *aw*e, respectively. Overall, participants did not assign different intensities to the top three-ranked emotions, $\chi 2(2) = 4.84$, $p = .09$. Kendall'*s* $W= .047$.

"Light Yellow". *Calmness* was the top ranked emotion for the color "light yellow", followed by *empathy*, and *happy*, respectively. Overall, participants did not assign different intensities to the top three-ranked emotions, $\chi 2(2) = 3.54$, $p = .17$. Kendall'*s* $W = .009$.

"Orange". *Alert* was the top ranked emotion for the color "orange", followed by *surprise* and a*nger,* respectively. Overall, participants did not assign different intensities to the top three-ranked emotions, $\chi 2(2) =2.73$, $p = .26$. Kendall'*s* $W = .026$.

"Pink". *Love* was the top ranked emotion for the color "pink", followed by *joy*, and *empathy*, respectively. Overall, participants did not assign different intensities to the top three-ranked emotions, $\chi 2(2) = 2.14$, $p = .34$. Kendall's $W = .021$.

"Purple". *Empathy* was the top ranked emotion for the color purple, followed by s*urprise*, and c*alm*ness, respectively. Overall, participants did not assign different intensities to the top three-ranked emotions, $\chi 2(2) = 1.17$, $p = .56$. Kendall's $W= .011$.

"Teal". *Calmness* was the top ranked emotion for the color "teal", followed by *empathy*, and *awe*, respectively. Overall, participants did not assign different intensities to the top three-ranked emotions, $\chi 2(2) = 2.18$, $p = .34$. Kendall's $W = .021$.

"Periwinkle". *Calmness* was the top ranked emotion for the color "periwinkle", followed by *sadness*, and *disappointment*, respectively. Overall, participants assigned different "periwinkle" color intensities to the top three-ranked emotions, $\chi 2(2) = 6.44$, $p = .04$. However,

the intensities assigned to *calmness* did not differ from those assigned to *sadness* (p = .20), and agreement was low. Kendall's W = .062

"Red". *Anger* was the top ranked emotion for the color "red", followed by *love*, and *alert*, respectively. Overall, participants assigned different intensities to the top three-ranked emotions, $\chi 2(2) = 13.92$, $p = .001$. However, the intensities assigned to anger did not statistically differ from the intensities assigned to *love*, $p = .10$, and surprisingly the agreement was low. Kendall's $W = .134$.

"White". *Boredom* was the top ranked emotion for the color "white", followed by *alert* and *calmness*. Overall, participants assigned different intensities to the top three-ranked emotions, $\chi 2(2) = 7.10$, $p = .03$. However, the intensities assigned to boredom did not significantly differ from alert, $p = .24$, and agreement was low. Kendall's $W = .068$.

"Yellow". *Alert* was the top ranked emotion for the color "yellow", followed by *happiness* and *joy.* Overall, participants did not assign different intensities to the top three-ranked emotions, $\chi 2(2) = 1.37$, $p = .51$. Kendall's $W = .013$.
