# Supplementary material for: What Color Is Your Anger? Assessing Color-Emotion Pairings in English Speakers
Source: Front Psychol. 2019 Feb 26;10:206. doi: 10.3389/fpsyg.2019.00206 (PMC6399154; doi:10.3389/fpsyg.2019.00206)
Supplement: Supplementary file 2 [file Data_Sheet_2.pdf]

Appendix A
Color Stimuli Used in Studies 1 and 2

Color	Swatch	Study	R	G	B	H	S	L	*CIE L**	*CIE a**	*CIE b**	Hex	Previous Studies
Light Red		Both	255	121	127	357	100	74	66.88	51.82	20.85	#FF797F	
Red		Both	255	0	0	0	100	50	53.23	80.42	66.97	#FF0000	
Dark Red		Study 2	192	0	0	0	100	38	39.93	64.97	54.09	#C00000	
Light Orange		Study 2	246	187	92	37	90	66	79.56	11.84	54.71	#FF9900	
Orange		Both	255	153	0	36	100	50	72.26	30.51	76.87	#FF6600	
Chocolate		Study 1	204	102	0	30	100	40	54.39	36.01	62.40	#CC6600	
Brown		Study 2	158	65	7	23	92	32	39.70	36.20	47.90	#9E4107	Hanada, 2017
Light Yellow		Study 2	255	252	164	58	100	82	97.80	-9.20	42.20	#FFFCA4	Hanada, 2017
Yellow		Both	255	255	51	60	100	60	97.24	-20.38	87.02	#FFFF33	
Dark Yellow		Both	204	153	0	45	100	40	66.23	8.61	69.98	#CC9900	
Light Green		Both	153	204	0	75	100	40	76.04	-38.99	75.18	#99CC00	
Bright Green		Both	0	255	0	120	100	50	87.74	-85.89	82.71	#00FF00	
Green		Both	0	164	39	134	100	32	58.69	-59.95	50.83	#00A427	
Jade		Study 1	0	153	102	160	100	30	55.83	-46.66	16.85	#009966	
Dark Green		Study 2	60	106	73	137	28	33	40.80	-23.50	13.10	#3C6A49	Hanada, 2017
Aqua		Study 1	0	255	255	180	100	50	91.12	-47.74	-15.11	#00FFFF	
Light Blue		Study 2	125	253	255	181	100	75	92.70	-33.50	-14.70	#7DFDFF	Hanada, 2017
Blue		Both	0	153	204	195	100	40	59.11	-15.14	-36.36	#0099CC	
Sky Blue		Study 1	0	204	255	192	100	50	76.32	-24.04	-37.64	#00CCFF	
Teal		Study 2	58	205	190	174	60	52	74.90	-40.90	-4.50	#3ABCDBE	Hanada, 2017; Kaya and Epps, 2004
Turquoise		Study 1	0	153	153	180	100	30	57.16	-32.60	-10.32	#009999	
Bright Blue		Study 2	51	47	255	241	100	59	38.10	68.70	-100.58	#332FFF	Hanada, 2017; Kaya and Epps, 2004
Dark Blue		Study 2	13	31	163	233	85	35	22.71	45.30	-70.67	#0D1FA3	Hanada, 2017
Indigo		Study 1	51	0	153	260	100	30	22.06	55.47	-69.10	#330099	
Periwinkle		Study 2	105	121	212	231	55	62	53.50	19.50	-49.80	#6919D4	Hanada, 2017; Kaya and Epps, 2004
Light Purple		Both	204	153	255	270	100	80	71.45	38.41	-44.59	#CC99FF	
Purple		Study 2	158	108	182	281	34	57	53.40	33.70	-32.00	#9E6CB6	Hanada, 2017; Kaya and Epps, 2004
Violet		Study 1	102	51	153	270	50	40	32.90	43.10	-47.80	#663399	
Dark Purple		Study 2	98	33	108	292	54	27	26.00	40.90	-30.80	#62206C	Hanada, 2017
Bright Pink		Both	255	51	255	300	100	60	62.38	92.56	-58.49	#FF33FF	
Pink		Both	255	102	204	320	100	70	65.84	68.95	-24.49	#FF6633	
Dark Pink		Both	204	51	153	320	60	50	48.89	67.83	-21.52	#CC3399	
White		Both	255	255	255	0	0	100	100.00	0.42	-0.99	#FFFFFF	
Gray		Both	102	102	102	0	0	40	0.00	0.00	0.00	#000000	
Black		Both	0	0	0	0	0	0	43.19	0.21	-0.50	#666666	
